# Lenvatinib inhibits the growth of gastric cancer patient-derived xenografts generated from a heterogeneous population

**DOI:** 10.1186/s12967-022-03317-7

**Published:** 2022-03-07

**Authors:** John D. Karalis, Lynn Y. Yoon, Suntrea T. G. Hammer, Changjin Hong, Min Zhu, Ibrahim Nassour, Michelle R. Ju, Shu Xiao, Esther C. Castro-Dubon, Deepak Agrawal, Jorge Suarez, Scott I. Reznik, John C. Mansour, Patricio M. Polanco, Adam C. Yopp, Herbert J. Zeh, Tae Hyun Hwang, Hao Zhu, Matthew R. Porembka, Sam C. Wang

**Affiliations:** 1 Department of Surgery, University of Texas Southwestern Medical Center, Dallas, TX USA; 2 Children’s Research Institute, Departments of Pediatrics and Internal Medicine, University of Texas Southwestern Medical Center, Dallas, TX USA; 3 Department of Pathology, University of Texas Southwestern Medical Center, Dallas, TX USA; 4 Department of Artificial Intelligence and Informatics, Department of Immunology, Mayo Clinic, Jacksonville, FL USA; 5 Department of Surgery, University of Florida, Gainesville, FL USA; 6 Department of Internal Medicine, University of Texas at Austin, Austin, TX USA; 7 Department of Gastroenterology and Hepatology, University of Texas Southwestern Medical Center, Dallas, TX USA; 8 Department of Cardiovascular and Thoracic Surgery, University of Texas Southwestern Medical Center, Dallas, TX USA; 9 Department of Surgery, Division of Surgical Oncology, University of Texas Southwestern Medical Center, 5323 Harry Hines Boulevard, Dallas, TX 75390 USA

**Keywords:** Gastric cancer, Patient-derived xenograft, PDX, Lenvatinib, NSG mice, Nude mice

## Abstract

**Background:**

Lenvatinib is a multitargeted tyrosine kinase inhibitor that is being tested in combination with immune checkpoint inhibitors to treat advanced gastric cancer; however, little data exists regarding the efficacy of lenvatinib monotherapy. Patient-derived xenografts (PDX) are established by engrafting human tumors into immunodeficient mice. The generation of PDXs may be hampered by growth of lymphomas. In this study, we compared the use of mice with different degrees of immunodeficiency to establish PDXs from a diverse cohort of Western gastric cancer patients. We then tested the efficacy of lenvatinib in this system.

**Methods:**

PDXs were established by implanting gastric cancer tissue into NOD.Cg-*Prkdc*^*scid*^*Il2rg*^*tm1Wjl*^/SzJ (NSG) or *Foxn1*^*nu*^ (nude) mice. Tumors from multiple passages from each PDX line were compared histologically and transcriptomically. PDX-bearing mice were randomized to receive the drug delivery vehicle or lenvatinib. After 21 days, the percent tumor volume change (%Δv_tumor_) was calculated.

**Results:**

23 PDX models were established from Black, non-Hispanic White, Hispanic, and Asian gastric cancer patients. The engraftment rate was 17% (23/139). Tumors implanted into NSG (16%; 18/115) and nude (21%; 5/24) mice had a similar engraftment rate. The rate of lymphoma formation in nude mice (0%; 0/24) was lower than in NSG mice (20%; 23/115; *p* < 0.05). PDXs derived using both strains maintained histologic and gene expression profiles across passages. Lenvatinib treatment (mean %Δv_tumor_: -33%) significantly reduced tumor growth as compared to vehicle treatment (mean %Δv_tumor_: 190%; *p* < 0.0001).

**Conclusions:**

Nude mice are a superior platform than NSG mice for generating PDXs from gastric cancer patients. Lenvatinib showed promising antitumor activity in PDXs established from a diverse Western patient population and warrants further investigation in gastric cancer.

**Supplementary Information:**

The online version contains supplementary material available at 10.1186/s12967-022-03317-7.

## Introduction

Gastric cancer is the third-leading cause of cancer-related deaths worldwide and was responsible for an estimated 769,000 deaths in 2020 [1]. The overall 5-year survival rate for gastric cancer patients is only 32% in the United States, and novel therapies are urgently needed [2]. However, efforts to develop effective anticancer therapies have been impeded by a lack of preclinical models that recapitulate the heterogeneity of gastric tumors. One strategy to overcome this limitation is the generation of patient-derived xenografts (PDX), in which human tumors are propagated in immunodeficient mice. PDXs are a completely in vivo preclinical platform that bypasses growing the tumor cells in in vitro environments under highly artificial conditions. As a result, PDXs retain a high degree of the histologic, genomic, and transcriptomic characteristics of the human tumors from which they are derived [3–5]. PDXs also exhibit superior predictive values for clinical outcomes than traditional in vitro cell culture and cell line-derived xenografts [6]. While gastric cancer PDX models have been established by East Asian and European institutions, few PDXs have been generated from Black/African American or Hispanic patients [4, 7, 8]. This is a significant deficiency because gastric cancer biology, and response to novel therapies, has been shown to vary by patient race and ethnicity [9–14].

The strain of recipient mice that should be used to establish gastric cancer PDX has not been determined. Commonly used strains include NOD.Cg-*Prkdc*^*scid*^* Il2rg*^*tm1Wjl*^/SzJ mice (NSG) and *Foxn1*^*nu*^ (nude) mice [5, 15]. NSG mice are highly immunodeficient and lack mature T cells, B cells, and natural killer cells and have defective macrophages and dendritic cells, whereas nude mice only lack mature T cells [16]. In theory, highly immunosuppressed strains may allow for higher tumor engraftment rates. However, the use of immunodeficient mice to generate solid tumor PDXs is hampered by the growth of B cell lymphoma, the frequency of which may be affected by the degree of immunodeficiency of the recipient mice [4, 5].

Lenvatinib is a multitargeted tyrosine kinase inhibitor and inhibits vascular endothelial growth factor receptors 1–3 (VEGFR 1–3), fibroblast growth factor receptors 1–4 (FGFR 1–4), platelet-derived growth factor receptors α and β (PDGFR α-β), RET (rearranged during transfection), and c-kit. However, it most potently affects the VEGFR family, suggesting a dominant anti-angiogenic mechanism [17, 18]. Anti-VEGFR therapy does have a role in gastric cancer treatment; ramicirumab, which is an anti-VEGFR2 monoclonal antibody, is approved for second-line therapy in advanced gastric cancer [19]. Recently, lenvatinib showed promising efficacy when given in combination with pembrolizumab, an immune checkpoint inhibitor, for the treatment of patients with advanced gastric cancer in a phase II clinical trial in Japan [20]. However, lenvatinib has not been well studied in the gastric cancer context as a monotherapy [21]. Thus, additional preclinical and clinical data are needed to understand the effects of lenvatinib monotherapy in a diverse gastric cancer patient population.

In this study, we generated PDXs from Western gastric cancer patients of various racial and ethnic backgrounds. We used NSG or nude mice as recipients to perform a head-to-head comparison of their rates of engraftment and lymphomatous growth. Finally, we used our PDX model to evaluate the efficacy of lenvatinib.

## Materials and methods

### Sample acquisition

This study was approved by the University of Texas Southwestern Medical Center institutional review board. All patients with gastric adenocarcinoma who were treated at Parkland Memorial Hospital (Dallas, TX) and William P. Clements Jr. University Hospital (Dallas, TX) were approached for the study. All enrolled patients provided written informed consent.

Tumor tissue was obtained from patients by endoscopic biopsy, gastric resection, and biopsy of metastatic sites. Samples for PDX generation were immediately placed into sterile phosphate buffered saline and transported on ice to the mouse procedure facility for implantation. Samples for transcriptomic analysis were immediately placed in RNAlater (Invitrogen) for at least 24 h at 4 °C and then transferred to − 80 °C.

### Mice

All mice were handled in accordance with the guidelines of the institutional animal care and use committee at the University of Texas Southwestern Medical Center. NSG and nude mice were obtained from Jackson Laboratory.

### PDX generation, passage, storage, and reanimation

Samples obtained by esophagogastroduodenoscopy (EGD) biopsy (each 1–3 mm^3^), biopsy of metastatic sites (1–3 mm^3^), and surgical resection (3–5 mm^3^) were coated in Matrigel Matrix (Corning Life Sciences) and implanted into the subcutaneous space in the flanks of recipient mice. Tumors were passaged when they reached a maximum diameter of 1.5 cm or skin necrosis overlying the tumor was observed. We considered a PDX line to be established if the tumor grew to a diameter of at least 1.5 cm and grew after passage to a second recipient mouse. At passage, the tumor was excised from the subcutaneous tissue, divided, and then: (1) passaged into the flanks of a new mouse, (2) fixed in 4% paraformaldehyde for histologic evaluation, and (3) stored in 10% dimethyl sulfoxide (DMSO) + 90% fetal bovine serum and submerged in liquid nitrogen for future use. To reanimate frozen PDX tissue, the tissue was warmed in a 37 °C water bath for 60 s and then implanted in an identical manner as a fresh sample. After reanimation, mice were maintained the same way as mice with primarily implanted xenografts.

### Lenvatinib testing

Four PDX lines were selected for drug testing. When tumors reached a volume of 300–500 mm^3^, mice were randomized to receive either the drug delivery vehicle (DMSO mixed with corn oil) or lenvatinib (MedChemExpress; cat #HY-10981) 10 mg/kg daily by oral gavage. Tumor dimensions were measured at baseline and once weekly thereafter by a blinded observer. After 21 days, all mice were euthanized. Tumor volume was calculated using the formula (π/6) × length × width^2^.[22] The percent tumor volume change (%Δv_tumor_) from baseline of all tumors was calculated. Mice were weighed weekly.

### Histology, immunohistochemistry, and immunofluorescence

Tissue samples were fixed in 4% paraformaldehyde for 24 h and then embedded in paraffin. Sections were cut to 4 μm. All samples were stained with hematoxylin and eosin (H&E) for histologic examination. Tumor and PDX histology were evaluated in a blinded fashion by a board-certified pathologist with expertise in gastrointestinal malignancies (S.T.G.H.).

Antibodies used for immunohistochemistry (IHC) were mouse anti-human pan-cytokeratin AE1/AE3 monoclonal antibody (1:500 dilution; Santa Cruz Biotechnology; SC-81714), mouse anti-human CD20 monoclonal antibody (Agilent; IR60461-2), rabbit anti-human CD31 monoclonal antibody (1:50 dilution; Abcam; ab28364), rabbit anti-human Ki-67 antibody (1:1000 dilution; Abcam, ab15580). Terminal deoxynucleotidyl transfer dUTP nick end label (TUNEL) immunofluorescence (IF) was performed using the In Situ Cell Death Detection Kit, AP (Roche). Hoechst 33342, Trihydrochloride, Trihydrate (Invitrogen) was used for nuclear counterstaining.

### Quantification of intratumoral vascular density, tumor cell proliferation, and apoptosis

Blood vessel density was quantified using the hot spot method [23]. In brief, each slide was scanned under light microscopy at 40× magnification to identify the three areas of highest vascular density. Those three areas were then evaluated at 200× magnification and the number of vessels per 200× magnification field were counted. Any stained ring of endothelial cells surrounding a lumen or discrete cluster of endothelial cells was counted as a single vessel. Two tumors from each PDX line were evaluated. Ki-67-positive cells were manually counted in three 400× fields per tumor. The Ki-67 of two tumors per treatment arm was calculated, and their average value was reported. To quantify TUNEL-positive cells, each slide was scanned under fluorescent microscopy at 40× magnification to identify the three areas of highest TUNEL-positivity. Those three areas were then evaluated at 200× magnification and the number of apoptotic cells per 200× magnification field were manually counted. The TUNEL-positivity rate of two tumors per treatment arm was calculated, and their average value was reported. All quantification was performed in a blinded fashion.

### RNA sequencing and analysis

RNA was extracted using the RNeasy Mini Kit (Qiagen). Total RNA was quantified using the Ribogreen method using Victor X2 fluorometry (Life Technologies). RNA integrity was assessed using an Agilent Technologies 2100 TapeStation. Libraries were made with TruSeq Stranded Total RNA kits (Illumina) and sequenced on a NovaSeq 6000 with 2 × 150 base pair paired-end reads.

RNA-sequencing data was refined by Fast p v0.20.1 (default settings) such that Illumina universal adapters were trimmed and reads with lower quality bases, or noninformative reads, were filtered out. A total of 51.3 million paired-end reads were retained. All samples engrafted into mice were aligned into both human (hg19) and mouse reference assembly (m38) using STAR (v2.7.9, —twopassMode Basic). Each aligned read was examined to determine the organism of origin. Reads uniquely aligned to the mouse genome (i.e., with a higher matching score than the human genome) were removed. Then, the refined human reads were converted to transcriptome abundant matrix in the format of transcripts per kilobase million (TPM) via Salmon v1.5.2 with input parameters: ‘-I IU—validateMappings’). The Salmon reference genome sequence index was built on human reference transcript sequences (GRCm38.v29) with the gene model Gencode v29 (Ensembl 94).

### Statistical analysis

Categorical variables were presented as counts and compared with chi-square or Fisher’s exact tests. The %Δv_tumor_ between vehicle and lenvatinib-treated tumors was compared with Mann–Whitney U tests, and the same tests were used for comparisons between vehicle and lenvatinib-treated vessel counts. A *p* value of < 0.05 was considered statistically significant. Statistical computation was performed using GraphPad Prism (Version 9.1.0).

## Results

### Gastric cancer PDXs were generated from a diverse Western population

Tumor samples were collected from 139 gastric cancer patients from 2015 to 2020. The baseline characteristics of the patients are summarized in Table 1. The median patient age was 55 years (range: 25–89 years), and 65% of patients were male. 55% of samples were obtained from patients who self-identified as being of Hispanic origin, 19% were from Black/African American patients, 4% were from Asian patients, 1% was from an American Indian patient, and 22% were from non-Hispanic White patients. 56% of patients presented with locally advanced tumors (T_2-4_N_0_M_0_, T_any_N_1-3_M_0_) and 42% of patients presented with metastatic disease (T_any_N_any_M_1_). Only 2% of patients presented with local disease (T_1-2_N_0_M_0_). 46% of patient tumors were of Lauren diffuse-type histology, 40% were intestinal-type, and 14% were mixed-type.

**Table 1:** Patient demographic, clinical, and pathologic factors

Variable	n (%)
Gender	
Male	91 (65)
Female	48 (35)
Race/ethnicity	
Non-Hispanic White	30 (22)
Black	26 (19)
Hispanic	77 (55)
Asian	5 (4)
American Indian	1 (1)
Presenting stage	
Local	2 (2)
Locally advanced	78 (56)
Metastatic	59 (42)
Lauren classification	
Intestinal	47 (40)
Diffuse	54 (46)
Mixed	17 (14)
Unknown	21
Differentiation	
Well	1 (1)
Moderate	31 (23)
Poor	102 (76)
Unknown	5
*H. pylori*	
Negative	82 (73)
Positive	30 (27)
Unknown	27
HER2	
0–1+ or 2+ NAmp	72 (77)
3+ or 2+ & amplified	21 (23)
Unknown	46

A *HER2* score of 3+ or 2+ and fluorescent in situ hybridization (FISH) amplified was considered positive. A *HER2* score of 0–1+ or 2+ and FISH non-amplified was considered negative

Amp, fluorescence in situ hybridization (FISH) amplified; Namp, FISH non-amplified

Overall, samples from 17% (23/139) of patients successfully engrafted (Table 2). 10 PDX lines were generated from Black/African American patients, 9 from Hispanic patients, 3 from non-Hispanic White patients, and 1 from an Asian patient. Next, we investigated whether clinicopathologic characteristics of the patient tumor were associated with PDX engraftment. We found that tumors from Black patients engrafted at a higher rate than other racial/ethnic groups and that Lauren intestinal-type tumors engrafted at a higher rate than diffuse-type tumors. Engraftment rate was not associated with whether the sample was obtained from EGD, surgical resection, or biopsy of a metastatic site (Table 2).

**Table 2:** Patient demographic, clinical, and pathologic factors associated with PDX engraftment

Variable	n (%)		*P* value
	Engrafted (n = 23)	Not engrafted (n = 116)	
Gender			
Male	19 (83)	72 (62)	NS
Female	4 (17)	44 (38)	
Race/ethnicity			
Non-Hispanic White	3 (13)	27 (23)	< 0.05
Black/African American	10 (43)	16 (14)	
Hispanic	9 (39)	68 (59)	
Asian	1 (4)	4 (3)	
American Indian	0 (0)	1 (1)	
Presenting stage			
Local	0 (0)	2 (2)	NS
Locally advanced	13 (57)	65 (56)	
Metastatic	10 (43)	49 (42)	
Lauren classification			
Intestinal	14 (61)	33 (35)	< 0.01
Diffuse	3 (13)	51 (54)	
Mixed	6 (26)	11 (12)	
Unknown	0	21	
Differentiation			
Well	0 (0)	1 (1)	NS
Moderate	7 (30)	24 (22)	
Poor	16 (70)	86 (77)	
Unknown	0	5	
*H. pylori* infection			
Negative	15 (79)	67 (72)	NS
Positive	4 (21)	26 (28)	
Unknown	4	23	
*HER2* status			
0–1+ or 2+ NAmp	12 (75)	60 (78)	NS
3+ or 2+ Amp	4 (25)	17 (22)	
Unknown	7	39	
Tissue source^a^			
EGD biopsy	9 (39)	96 (54)	NS
Resection	8 (35)	56 (32)	
Metastasis biopsy	6 (26)	25 (14)	

A *HER2* score of 3+ or 2+ and fluorescent in situ hybridization (FISH) amplified was considered positive. A *HER2* score of 0–1+ or 2+ and FISH non-amplified was considered negative

Amp, fluorescence in situ hybridization (FISH) amplified; EGD, esophagogastroduodenoscopy; NAmp, FISH non-amplified

^a^Multiple samples were collected from some patients

### Gastric cancer PDXs are histologically and transcriptomically similar to the parent tumors from which they were derived

**Fig. 1:**
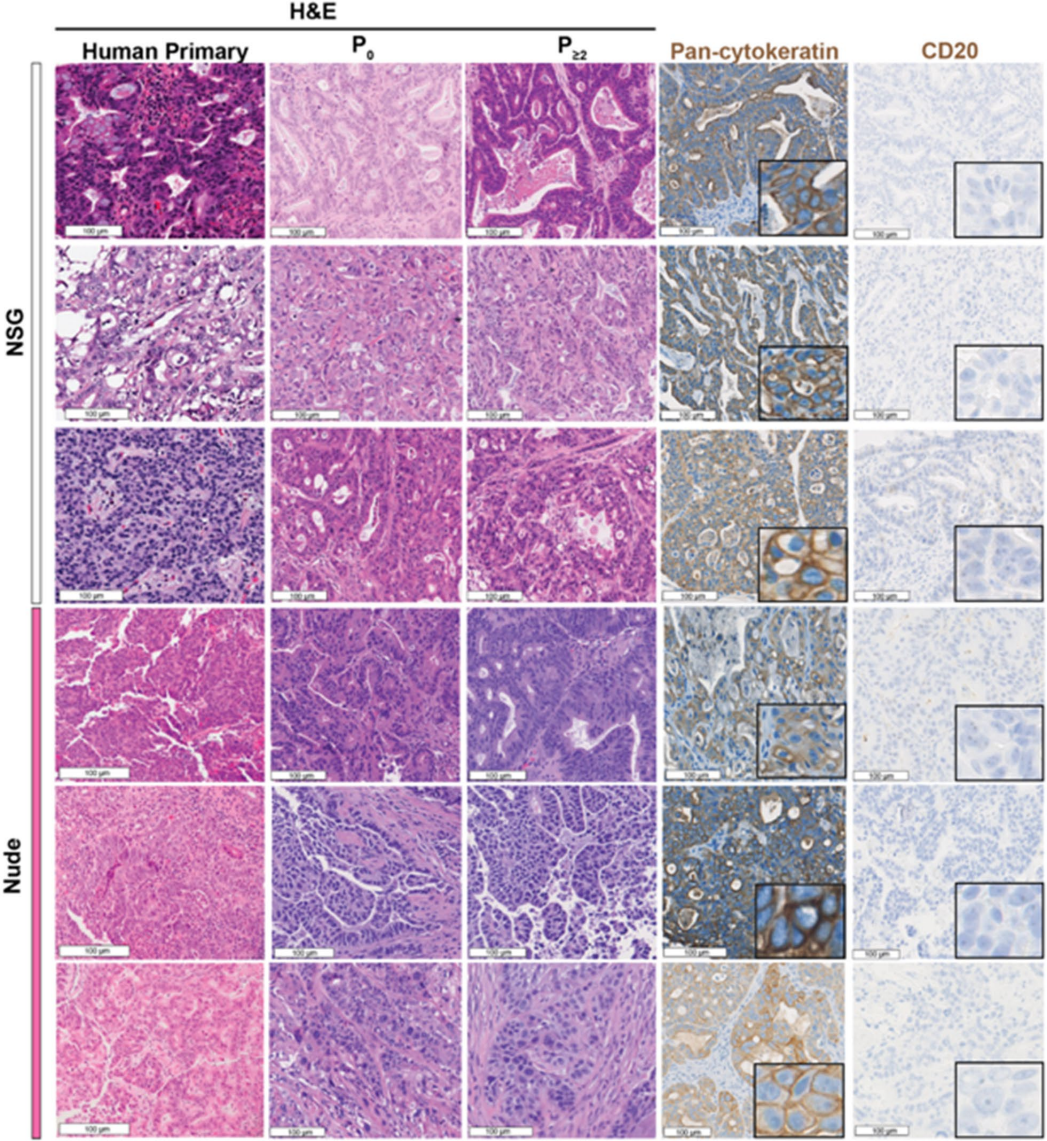
Gastric cancer PDXs maintain histologic characteristics over passages. Columns (left to right) depict: H&E of the primary tumor, H&E of the first engrafted PDX tumor (denoted as P0), H&E of the PDX after at least 2 passages in mice (denoted as P ≥ 2), immunohistochemistry (IHC) for pan-cytokeratin, IHC for CD20. insets at 400× magnification

**Fig. 2:**
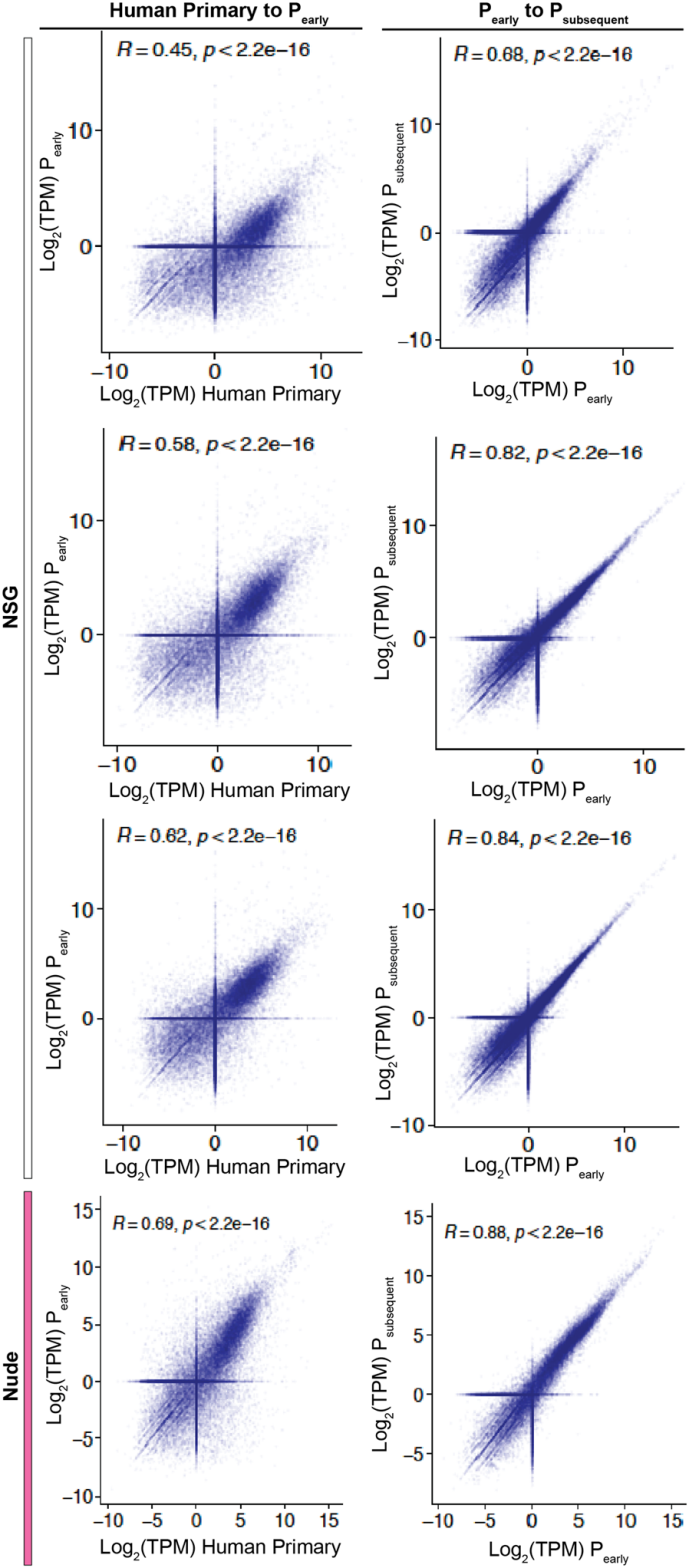
Gastric cancer PDXs maintain stable gene expression profiles over passages**.** Tumor gene expression profiles were analyzed by bulk RNA-sequencing. Comparisons were made between the human primary tumor and an early-passage PDX, as well as between an early passage PDX and a subsequent PDX passage. Transcripts per kilobase million (TPM) values were transformed to log_2_(TPM + 1). The Pearson correlation coefficient (*R*) and p-values are annotated

Engrafted PDXs retained the histologic characteristics of the human primary tumors they were derived from, including after the PDX was passaged, regardless of whether the PDX was propagated in NSG or nude mice (Fig. 1). We also compared the transcriptomic profiles of the tumors via bulk RNA sequencing. We first analyzed the similarity of gene expression between the human primary tumor and an early passage PDX. We found a modest correlation (Pearson correlation coefficient 0.45–0.69, Fig. 2); however, this was expected because the human primary tumor sample also included cells of the human tumor microenvironment such as fibroblasts and immune cells; whereas the PDX tumor, due to its growth in an immunodeficient mouse, has fewer infiltrating immune cells. We then compared an early passage PDX with a subsequently passaged PDX and found a strong correlation between their gene expression profiles (Pearson correlation coefficient 0.68–0.88, Fig. 2) which indicates that our PDX model exhibits a high degree of transcriptomic stability, after multiple passages. Notably, we found similar stability of gene expression between PDXs propagated in NSG and nude mice.

**Fig. 3:**
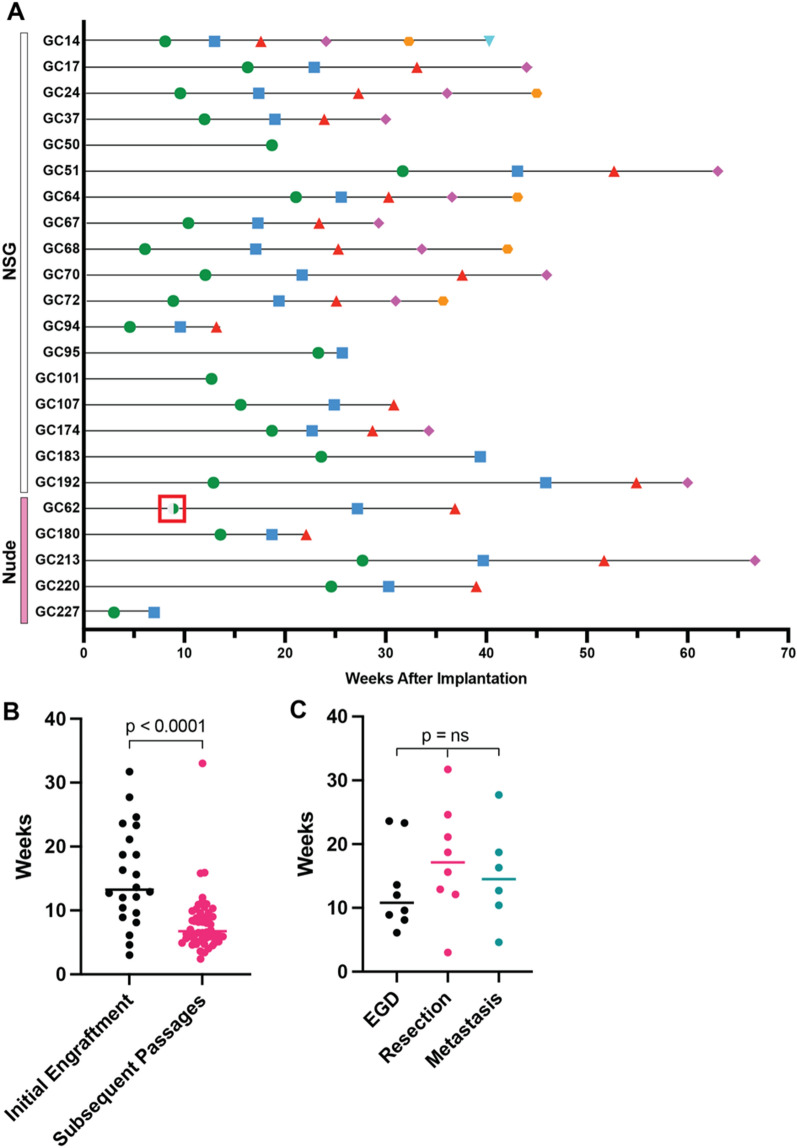
**A** Time to initial PDX engraftment (green circle) and all subsequent passages. The blue square indicates the time that the second PDX passage engrafted. The red triangle, purple diamond, orange hexagon, and upside-down teal triangle, indicate the engraftment time of the third, fourth, fifth, and sixth PDX passages, respectively. The half-filled green circle surrounded by a red box (GC62) indicates a tumor comprised of gastric adenocarcinoma and lymphoma at a 1:1 ratio, initially implanted into an NSG mouse and subsequently propagated in nude mice. **B** Median time to initial PDX engraftment versus all subsequent passages. **C** Median time to engraftment by sample source. EGD, esophagogastroduodenoscopy

The median time to initial PDX engraftment was 13.3 weeks while the median number of weeks between subsequent passages was significantly lower at 6.8 weeks (*p* < 0.0001; Fig. 3A, B). There was no difference in time to engraftment based on whether the tissue was obtained by EGD, surgical resection, or biopsy of a metastatic site (Fig. 3C). All PDXs stored in liquid nitrogen were able to be successfully reanimated, even after more than 5 years.

### Nude mice are a better recipient than NSG mice for PDX generation

We first tested NSG mice as recipients for PDXs. We found that while 36% (41/115) of samples resulted in tumor growth, 56% (23/41) of the tumors were lymphomas, as confirmed by H&E staining, negative pan-cytokeratin AE1/AE3 IHC, and positive CD20 IHC. The remaining 18 tumors were histologically consistent with gastric adenocarcinoma and were pan-cytokeratin AE1/AE3-positive, and CD20-negative. Thus, the rate of successful PDX generation in NSG mice was 16% (18/115). We then tested nude mice as the recipient platform and found a similarly successful PDX establishment rate of 21% (5/24; Table 3). There was no difference in median time to initial gastric cancer engraftment between the strains (NSG: 12.8 weeks, nude: 19.1 weeks; *p* = NS). Importantly, we observed no lymphomas in nude mice. The difference in lymphoma formation in NSG (20%; 23/115) and nude mice (0%; 0/24) was significant (Table 3; *p* < 0.05).

**Table 3:** Comparison of NSG and nude mice as recipients for gastric cancer PDX generation

	n (%)		*P* value
	NSG (n = 115)	Nude (n = 24)	
Gastric adenocarcinoma	18 (16)	5 (21)	NS
Lymphoma	23 (20)	0 (0)	< 0.05

### Transplant of a mixed gastric cancer-lymphoma PDX from an NSG to a nude mouse eradicated lymphoma

**Fig. 4:**
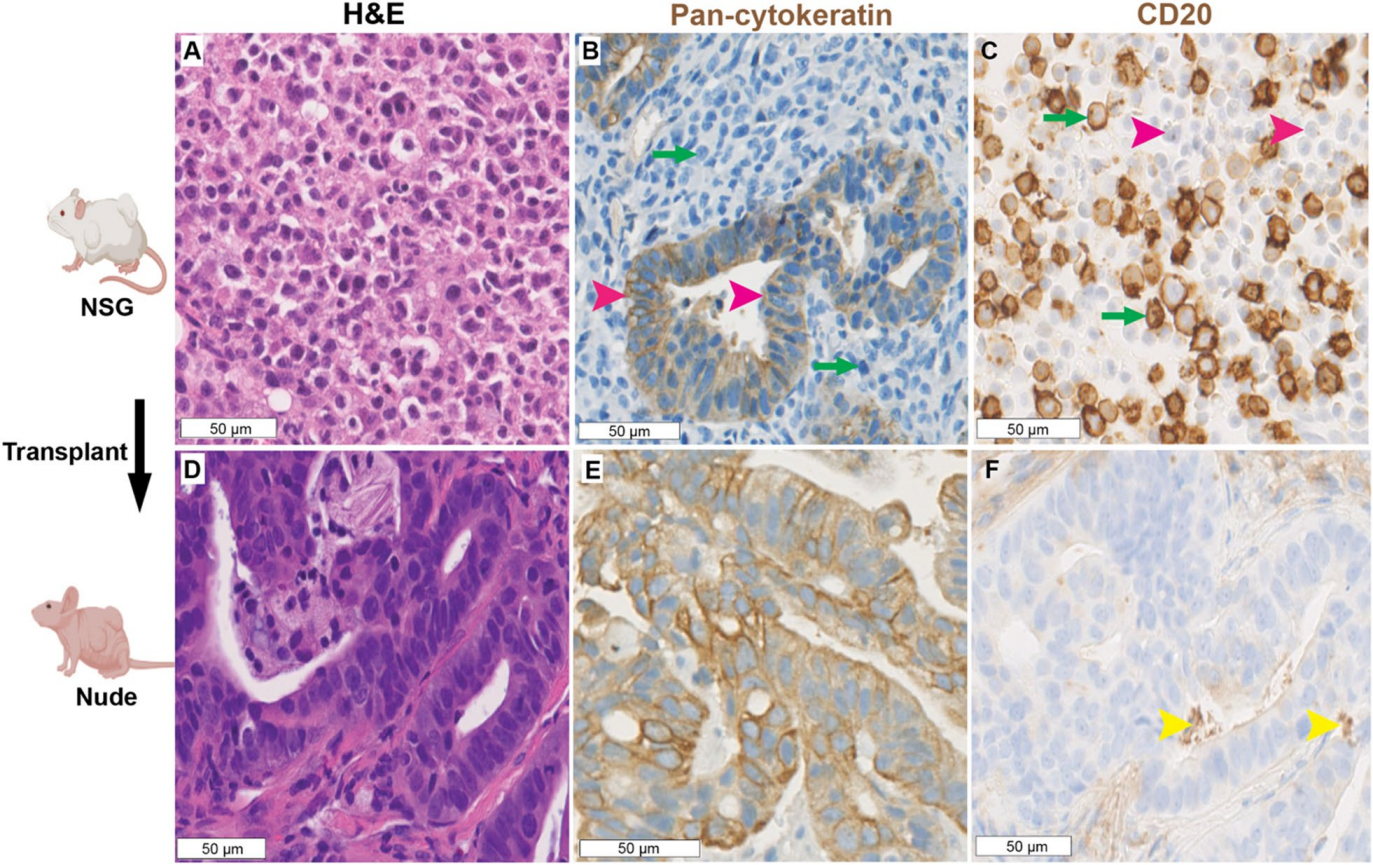
Mixed gastric adenocarcinoma-lymphoma PDXs can be “rescued” by propagation in nude mice. The PDX was initially established in NSG mice and contained both gastric adenocarcinoma and B-cell lymphoma as shown by **A** H&E, **B** pan-cytokeratin IHC (pink arrowheads to positive staining gastric adenocarcinoma cells, green arrows to negative staining lymphoma cells), and **C** CD20 IHC (pink arrowheads to negative staining gastric adenocarcinoma cells, green arrowheads to positive staining lymphoma cells). After transplantation to nude mice, the tumor contained only gastric adenocarcinoma as confirmed by **D** H&E, **E** pan-cytokeratin IHC, and **F** CD20 IHC (yellow arrowheads show non-specific staining in glandular lumens)

One sample implanted into an NSG mouse resulted in a tumor that contained both gastric adenocarcinoma and lymphoma in an approximate 1:1 ratio (Fig. 4A). The presence of a mixed population was confirmed with pan-cytokeratin and CD20 IHC (Fig. 4B, C). Since we had previously found that no lymphomas developed in nude mice, we hypothesized that we could “rescue” the gastric adenocarcinoma component by propagating the mixed tumor in nude mice. We passaged the tumor into nude mice and analyzed the tumor that formed. We found that the tumor histology was consistent with gastric adenocarcinoma and no lymphoma was observed (Fig. 4D). All tumor cells were pan-cytokeratin positive (Fig. 4E) and there were no CD20-positive cells (Fig. 4F). These histological findings were maintained on subsequent passages. Overall, these data support the notion that the lymphomatous component was eradicated upon passaging in nude mice.

### Lenvatinib inhibited the growth of gastric cancer PDXs and reduced intratumoral vascular density

**Fig. 5:**
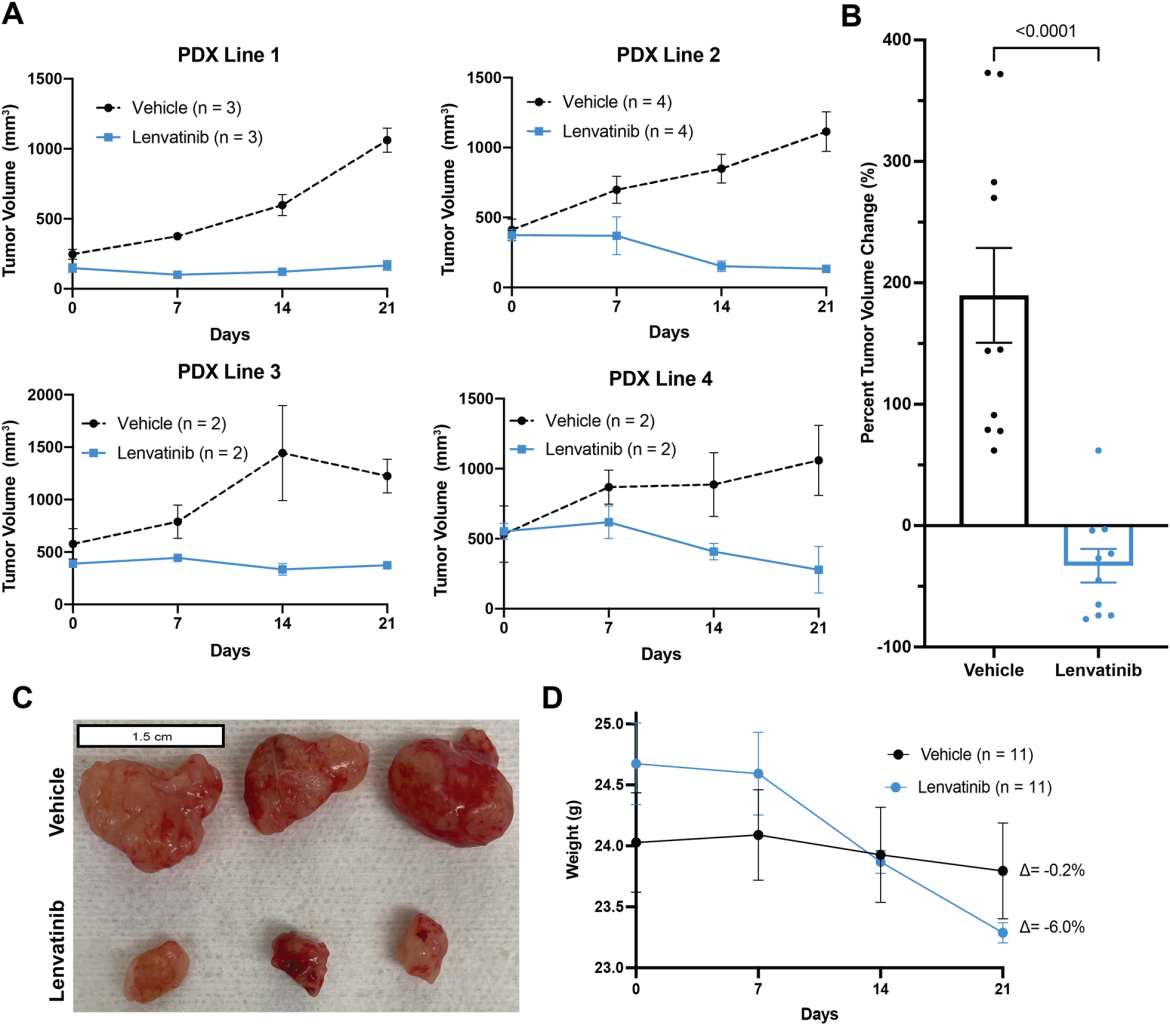
Gastric cancer PDXs respond robustly to lenvatinib. **A** Growth curves for the vehicle and lenvatinib-treated PDX lines. **B** Percent change in tumor volume of the vehicle and lenvatinib-treated PDX lines. Each dot represents one mouse. The error bar represents the standard error of the mean. **C** Vehicle and lenvatinib-treated tumors from PDX Line 1. **D** Percent change of mouse body weight throughout treatment

We then evaluated the utility of our PDX model for preclinical drug testing. Four PDX lines with heterogeneous features were selected for testing. These PDX lines were chosen to represent patients from multiple racial/ethnic groups, Lauren diffuse- and intestinal-type tumors, chemotherapy-treated and chemotherapy-naïve tumors, as well as both gastric and gastroesophageal junction tumors (Additional file 1: Table S1). Lenvatinib inhibited the growth of all four unique PDX lines (%Δv_tumor_ range: − 2% to − 63%). An analysis of all vehicle (n = 11) and lenvatinib-treated (n = 11) PDXs yielded a mean %Δv_tumor_ of 190% and − 33%, respectively (*p* < 0.0001; Fig. 5A–C). Vehicle-treated mice had an average change in body weight of 0.2 g (− 1%) and lenvatinib-treated mice had an average change of − 1.4 g (− 6.0%; *p* < 0.05; Fig. 5D). No unscheduled euthanasia was required.

**Fig. 6:**
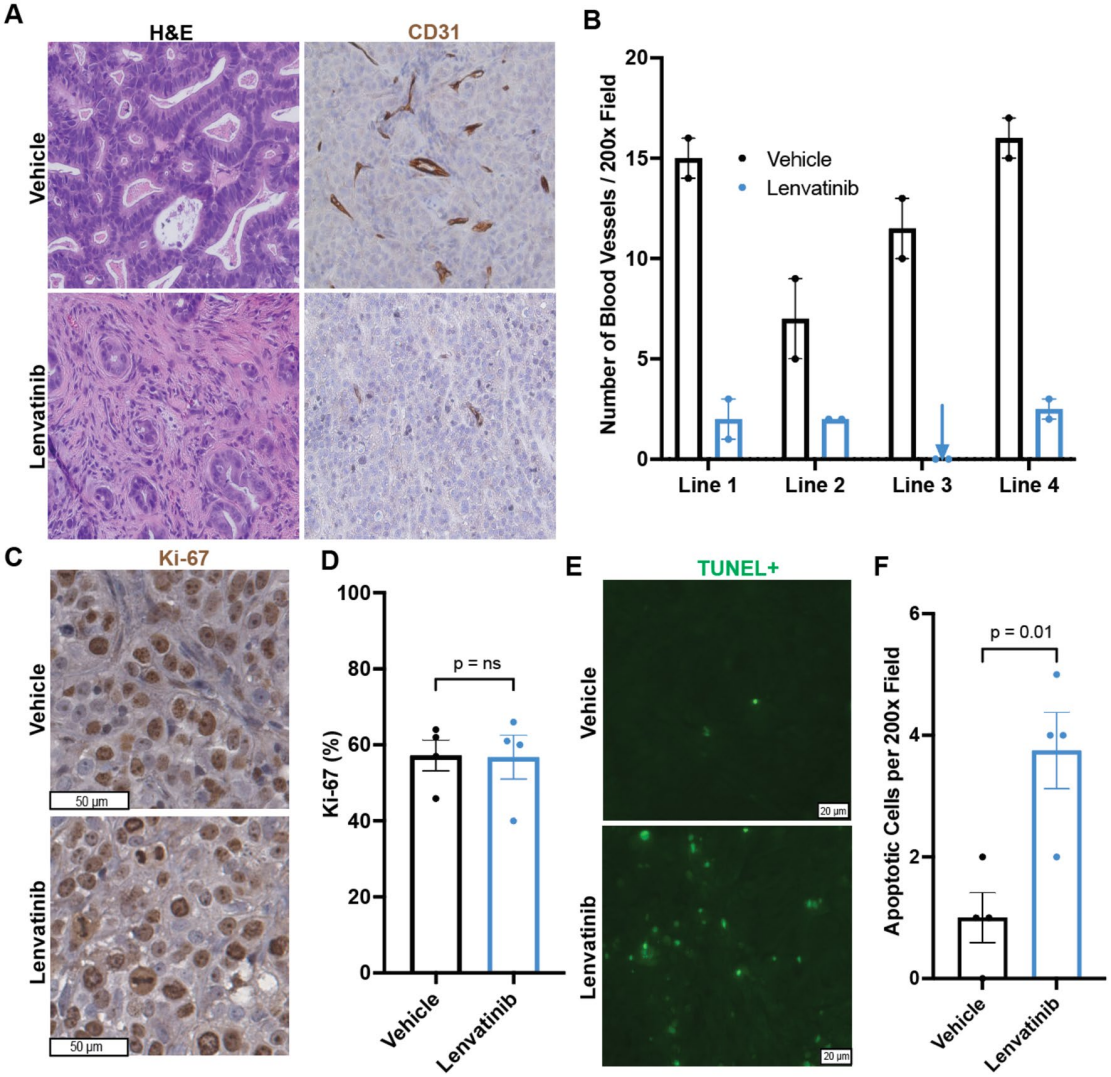
Lenvatinib treatment reduced blood vessel density and increased apoptosis in PDX tumors. **A** H&E and CD31 IHC of vehicle and lenvatinib-treated PDXs (200× magnification) **B** Intratumoral vascular density quantification. Each dot represents one tumor. The error bar represents the standard error of the mean. **C** Ki-67 staining of vehicle and lenvatinib-treated tumors. **D** Ki-67 quantification of vehicle and lenvatinib-treated tumors. Each dot represents the average Ki-67 percentage per PDX line. The error bar represents the standard error of the mean. **E** TUNEL immunofluorescence staining of vehicle and lenvatinib-treated tumors. **F** Quantification of TUNEL-positive cells. Each dot represents the average TUNEL-positive cells per PDX line. The error bar represents the standard error of the mean

H&E examination of vehicle and lenvatinib-treated tumors revealed markedly increased tumor necrosis and desmoplasia in the lenvatinib-treated samples (Fig. 6A). Because the dominant action of lenvatinib is VEGF receptor inhibition, which would impede tumor angiogenesis, we next performed IHC directed at the endothelial cell marker CD31. The average number of vessels per 200 × field in the vehicle group was 12.4 (range: 7–16) versus 1.6 (range: 0–2.5) in the lenvatinib-treated group (*p* < 0.05; Fig. 6A, B), thus confirming that lenvatinib therapy had the on-target effect of reducing tumor vasculature. We next determined the effect of lenvatinib treatment on tumor cell proliferation and apoptosis. We found no difference in Ki-67 percentage between vehicle and lenvatinib-treated tumors (Fig. 6C, D). However, we did find a significantly increased number of apoptotic cells in lenvatinib-treated tumors (Fig. 6E, F).

## Discussion

In this study, we established 23 gastric cancer PDX lines from patients of diverse racial and ethnic backgrounds. PDXs are unique among preclinical models in that they recapitulate the native tumor biology of the patients from whom they are derived. However, to date, gastric cancer PDXs have primarily been generated from European and East Asian patients [4, 7, 8]. The lack of diversity in previously generated gastric cancer PDXs is a major shortcoming for the field as there is significant biological heterogeneity associated with patient race and ethnicity [9–14]. For example, Strong et al. [9] found that patients in Korea had improved gastric cancer-specific survival when compared to patients in the US, even after controlling for other prognostic variables. Additionally, differing races and ethnicities have exhibited divergent responses to therapy; for example, in a phase II study of regorafenib, Asian patients were found to have a more favorable response than patients of other races and ethnicities [11]. To our knowledge, this is the first description of PDXs generated from a large number of Black/African American and Hispanic patients.

The preferred recipient mouse strain for PDX generation has not been established [4, 5]. Theoretically, a highly immunodeficient mouse strain, such as the NSG line that lacks mature T cells, B cells, and natural killer cells, may allow for a higher tumor engraftment rate, but at a cost of increased lymphomatous growth as compared to a modestly immunodeficient strain, such as nude mice, which lack only mature T cells [16]. To answer this question, we performed a head-to-head comparison of NSG and nude mice as recipients. We found that while engraftment rates were similar between the two strains, there was a lower rate of lymphoma formation in nude mice. The unregulated proliferation of xenografted patient B cells leads to lymphoma formation in immunodeficient mice. Our findings demonstrate that the presence of some functional elements of the immune system in nude mice is sufficient to suppress lymphoma overgrowth without affecting the rate of gastric adenocarcinoma engraftment. Moreover, we found that PDXs engrafted into NSG and nude mice similarly retained the histologic and transcriptomic characteristics of the human primary tumor from which they were derived. While administration of the anti-CD20 antibody rituximab has been suggested as a method to reduce lymphomatous transformation in gastric cancer PDXs, we have not found this to be necessary due to the success of our nude mouse protocol [15, 24]. Thus, our results demonstrate that nude mice are a preferred platform over NSG mice for gastric cancer PDX generation. Our findings are consistent with the report by Choi et al. who also concluded that nude mice are the preferred recipient for gastric cancer PDX generation [5]. This has significant logistical implications as nude mice are less expensive than NSG mice and may have fewer housing and handling restrictions.

The efficacy of novel therapies in the preclinical setting is infrequently replicated in clinical trials [25]. This may be explained, in part, by the degree to which preclinical research relies on cancer cell lines that are established as a monolayer growing in a nutrient-rich environment. In contrast, PDXs are unmodified by in vitro adaptation and have been shown to maintain a high degree of the histologic, genomic, and transcriptomic features of the parent tumors from which they were derived [5, 26]. As a result, PDXs are a demonstrably better platform for preclinical drug testing [27].

We found that lenvatinib inhibited the growth of all four PDX lines tested, regardless of patient race/ethnicity, Lauren subtype, histologic differentiation, anatomic tumor location, and history of prior chemotherapy treatment. This suggests that lenvatinib monotherapy may be effective against a broad range of gastric cancers, although there is a paucity of data in gastric cancer patients. Lenvatinib has only been tested as a monotherapy in 6 advanced gastric cancer patients in a phase I study in which 3 of the 6 patients showed stable disease [21]. In the KEYNOTE-061 trial, the objective response rate of gastric cancer patients treated with pembrolizumab was approximately 15% in patients with a combined positive score (CPS) of ≥ 1 [28]. In contrast, Kawazoe et al.’s findings from a phase II trial that tested a combination of lenvatinib plus pembrolizumab showed an objective response in 69% of patients, despite only 66% of the patient cohort having a CPS of ≥ 1 [20]. As such, we reasoned that in the Kawazoe et al. cohort, either: (1) lenvatinib was the primary driver of the increased response rates observed, or (2) lenvatinib enhanced the efficacy of pembrolizumab. Because we found that lenvatinib was effective in the absence of concurrent immune checkpoint inhibition, our data suggest that lenvatinib may, at least in part, be an independent driver of the results observed in the Kawazoe et al. study. Another notable finding was the efficacy of lenvatinib in all three treatment-naïve PDX lines. This is significant because ramucirumab, an anti-angiogenic monoclonal antibody that blocks VEGFR-2, has demonstrated efficacy only in a second-line setting [29]. The broader range of lenvatinib’s anti-angiogenic inhibition via blockade of VEGFR1-3 may be more effective for the treatment of gastric cancer than selective VEGFR-2 inhibition that ramucirumab provides.

There are several limitations to our study. There is interest in using PDXs as a “patient avatar” to test therapies and identify the treatment options on an individualized basis. However, we were only able to generate PDXs from approximately 1 in 6 patients and our median time to engraftment was 13 weeks. Therefore, while this personalized approach is attractive in principle, it may not be feasible at most centers. Additionally, PDX-bearing mice are immunocompromised; as such, tumor-immune interactions and immune-oncologic therapies cannot be easily evaluated in PDXs. Finally, the influence of the tumor microenvironment in the subcutaneous space of the mouse flank is different than influences from gastric tissue and the unique environment of the stomach lumen. Despite these limitations, we believe that PDXs are a robust model for generating high-quality preclinical data for novel gastric cancer therapies.

## Conclusion

In this report, we describe the successful establishment of a PDX model from a diverse gastric cancer patient population. We recommend nude mice as the preferred recipient strain over NSG mice for gastric cancer PDX generation as we found lymphomas were unable to grow in nude mice. Finally, lenvatinib is a promising agent for the treatment of gastric cancer and warrants further investigation in clinical trials beyond in combination with pembrolizumab.

## Supplementary Information

**Additional file 1: Table S1.** Patient demographic, clinical, and pathologic characteristics of lenvatinib-treated PDXs. Abbreviation: GEJ, gastroesophageal junction.

## Data Availability

The datasets used and/or analyzed during the current study are available from the corresponding author on reasonable request.
