# Correction to: Lenvatinib inhibits the growth of gastric cancer patient‑derived xenografts generated from a heterogeneous populations

**DOI:** 10.1186/s12967-024-05632-7

**Published:** 2024-09-13

**Authors:** John D. Karalis, Lynn Y. Yoon, Suntrea T. G. Hammer, Changjin Hong, Min Zhu, Ibrahim Nassour, Michelle R. Ju, Shu Xiao, Esther C. Castro‑Dubon, Deepak Agrawal, Jorge Suarez, Scott I. Reznik, John C. Mansour, Patricio M. Polanco, Adam C. Yopp, Herbert J. Zeh III, Tae Hyun Hwang, Hao Zhu, Matthew R. Porembka, Sam C. Wang

**Affiliations:** 1 Department of Surgery, University of Texas Southwestern Medical Center, Dallas, TX USA; 2 Children’s Research Institute, Departments of Pediatrics and Internal Medicine, University of Texas Southwestern Medical Center, Dallas, TX USA; 3 Department of Pathology, University of Texas Southwestern Medical Center, Dallas, TX USA; 4 Department of Artificial Intelligence and Informatics, Department of Immunology, Mayo Clinic, Jacksonville, FL USA; 5 Department of Surgery, University of Florida, Gainesville, FL USA; 6 Department of Internal Medicine, University of Texas at Austin, Austin, TX USA; 7 Department of Gastroenterology and Hepatology, University of Texas Southwestern Medical Center, Dallas, TX USA; 8 Department of Cardiovascular and Thoracic Surgery, University of Texas Southwestern Medical Center, Dallas, TX USA; 9 Department of Surgery, Division of Surgical Oncology, University of Texas Southwestern Medical Center, 5323 Harry Hines Boulevard, Dallas, TX 75390 USA

Following publication of the original article [1], we have been notified that Figure 6c was published incorrectly.

It is now:

**Fig. 6:**
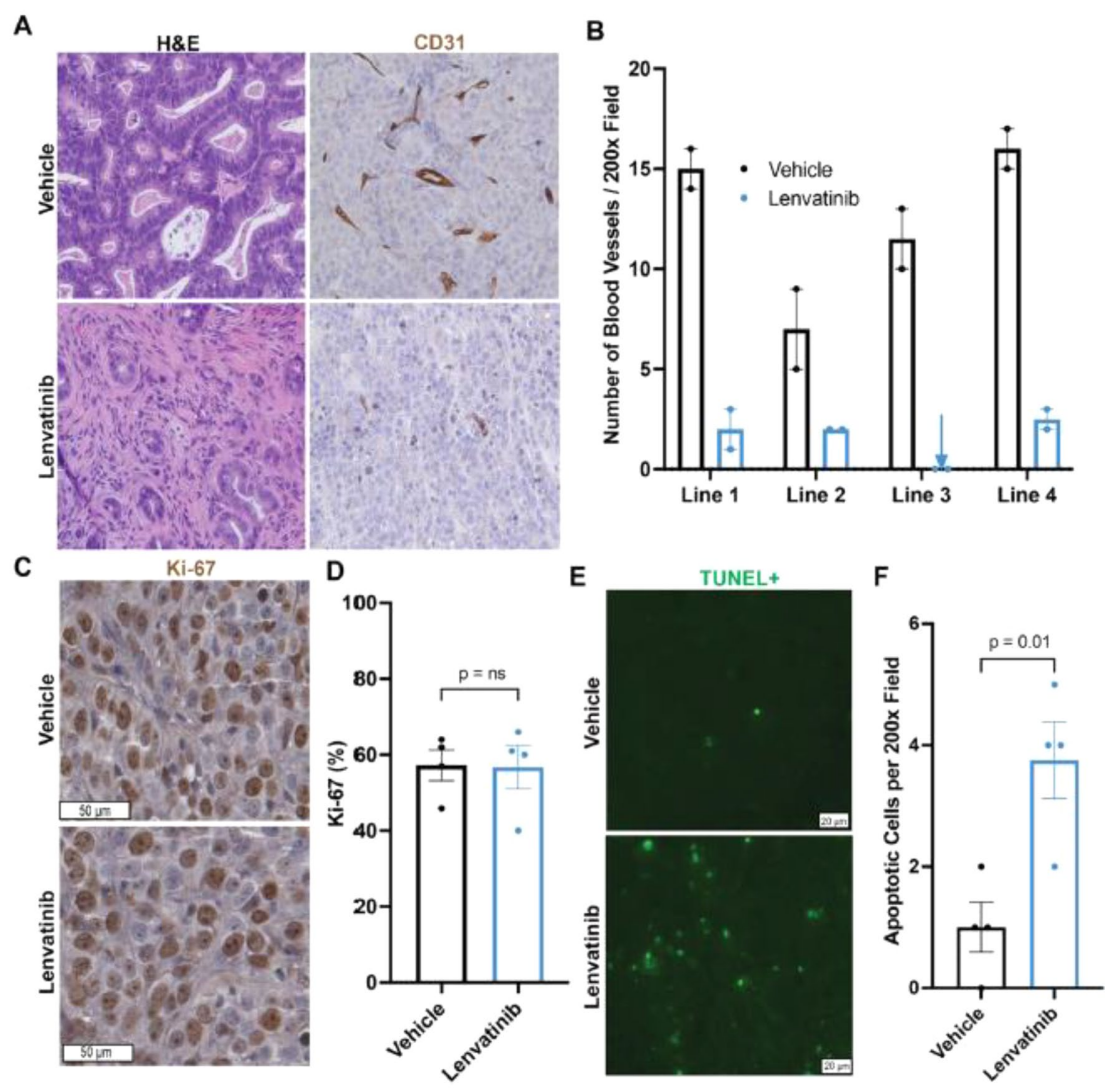
Lenvatinib treatment reduced blood vessel density and increased apoptosis in PDX tumors. **A** H&E and CD31 IHC of vehicle and lenvatinib-treated PDXs (200× magnification) **B** Intratumoral vascular density quantification. Each dot represents one tumor. The error bar represents the standard error of the mean. **C** Ki-67 staining of vehicle and lenvatinib-treated tumors. **D** Ki-67 quantification of vehicle and lenvatinib-treated tumors. Each dot represents the average Ki-67% per PDX line. The error bar represents the standard error of the mean. **E** TUNEL immunofluorescence staining of vehicle and lenvatinib-treated tumors. F Quantification of TUNEL-positive cells. Each dot represents the average TUNEL-positive cells per PDX line. The error bar represents the standard error of the mean

It should be:

**Fig. 6:**
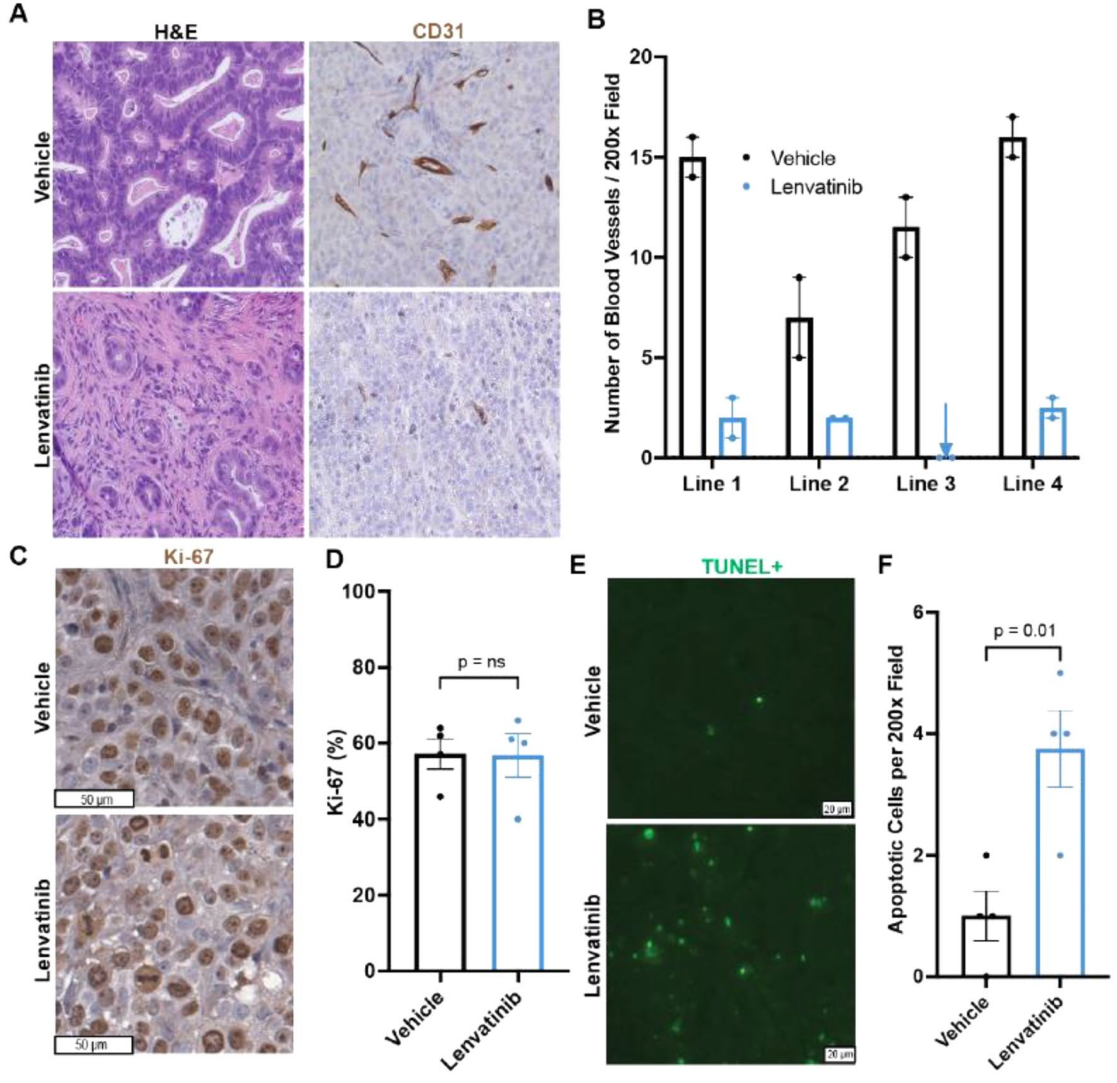
Lenvatinib treatment reduced blood vessel density and increased apoptosis in PDX tumors. **A** H&E and CD31 IHC of vehicle and lenvatinib-treated PDXs (200× magnification) **B** Intratumoral vascular density quantification. Each dot represents one tumor. The error bar represents the standard error of the mean. **C** Ki-67 staining of vehicle and lenvatinib-treated tumors. **D** Ki-67 quantification of vehicle and lenvatinib-treated tumors. Each dot represents the average Ki-67% per PDX line. The error bar represents the standard error of the mean. **E** TUNEL immunofluorescence staining of vehicle and lenvatinib-treated tumors. F Quantification of TUNEL-positive cells. Each dot represents the average TUNEL-positive cells per PDX line. The error bar represents the standard error of the mean

The original article was updated.
