# Supplementary material for: Lenvatinib inhibits the growth of gastric cancer patient-derived xenografts generated from a heterogeneous population
Source: J Transl Med. 2022 Mar 7;20:116. doi: 10.1186/s12967-022-03317-7 (PMC8900296; doi:10.1186/s12967-022-03317-7)
Supplement: Supplementary file 1 — Additional file 1: Table S1. Patient demographic, clinical, and pathologic characteristics of lenvatinib-treated PDXs. Abbreviation: GEJ, gastroesophageal junction. [file 12967_2022_3317_MOESM1_ESM.docx]

**Additional file 1: Table S1.** Patient demographic, clinical, and pathologic characteristics of lenvatinib-treated PDXs. Abbreviation: GEJ, gastroesophageal junction

| **PDX line** | **Age** | **Gender** | **Race** | **Ethnicity** | **Presenting stage** | **Anatomic location** | **Differentiation** | **Lauren classification** | **Prior chemotherapy** |
| --- | --- | --- | --- | --- | --- | --- | --- | --- | --- |
| **1** | 57 | M | White | Hispanic | Middle | GEJ | Moderate | Intestinal | No |
| **2** | 52 | M | White | Non-Hispanic | Middle | GEJ | Moderate | Intestinal | No |
| **3** | 51 | M | Black | Non-Hispanic | Late | Gastric | Poor | Diffuse | No |
| **4** | 45 | F | Black | Non-Hispanic | Late | Gastric | Poor | Intestinal | Yes |
